# Commentary on: Effects of Lumbar Discectomy on Disability and Depression in Patients with Chronic Low Back Pain

**DOI:** 10.5812/kowsar.22287523.2118

**Published:** 2011-09-26

**Authors:** Leszek Herbowski

**Affiliations:** 1 Neurosurgery and Neurotraumatology Department, District Hospital, Szczecin, Poland

**Keywords:** Low back pain, Depression, Laminectomy

*Dear Editor*,

My objective was to explore and analyze the influence of depression on the change in disability index in patients undergoing lumbar spine surgery on the basis of a work presented by Farzanegan, et al. (1).

It is well known in the pain and psychiatric literature that chronic low back pain (LBP) is commonly associated with depression and disability. However it is not well understood if the pain is accompanied by depression (secondary or reactive depression) or the pain is a consequence of depression (primary depression). It is assumed that about between 20% and 45% of patients with chronic pain are diagnosed to be depressed (2, 3). Multivariate regression analyses found that failure to return to work was predicted due to presurgical depression (P < 0. 01) and failure to report improved functional abilities was predicted due to presurgical somatic depression (P < 0. 05) (4).

Therefore, it would be interesting to find out the outcome results when two patients have low back pain and depression, but only one of them is being treated. The answer can be found in a paper by Farzanegan et al. (1) reporting the study where 148 patients with LBP and depression had laminectomy procedure. This study broadens an understanding of the need to improve the patient-oriented assessment of spinal surgery outcomes, especially in the range of function state and depressive symptoms. The physical disability was screened with the use of Rolland and Morris’s 24-item questionnaire. The scores of Rolland and Morris’s scale cover some daily activities related to pain and thus this tool is appropriate for patients with chronic pain. Severity of depression was assessed by Beck Depression Inventory (BDI). This 21-item diagnostic scale is widely used to identify minimal, mild, moderate or severe depression. The authors in the study used 9 as a cut-off point. The main result of the study is that depression and disability scores of patients decreased significantly after lumbar laminectomy. Six and 12 months after laminectomy the mean disability-score improvement was 58. 2 ± 21. 8 and 74. 69 ± 28. 12 respectively. Additionally, the mean of depression improvement score in female and male participants was 9. 6 ± 13. 92 and 2. 7 ± 12. 7 respectively. On the basis of the data presented depression prevalence ranged from 69. 4% among the patients before surgery to 49. 7% and 44. 2% 6 and 12 months after laminectomy respectively. This is much more than in a report by Sinikallio in which depression incidence was estimated at the level of 20% among Finnish patients before lumbar spinal stenosis surgery (5). In a cross-national study by Demyttenaere et al. major depression prevalence among patients with back pain ranged between 2. 5% (Lebanon) and 15. 7% (United States) (2). Firstly, the greater prevalence of depression in the current study may be the country-related medical specificity. Secondly, the higher incidence of both disorders among all patients is probably the consequence of lowering the cut-off point in the current study. According to Lasa et al. the optimal Beck Depression Inventory cut-off score is 13 (sensitivity 100%, specificity 99%) (6). Furthermore, Sinikallio et al. used the Beck Depression Inventory cut-off amounting to 14 (sensitivity 88%, specificity 84%) and they stated that the higher cut-off score was set not to overestimate depression occurrence (5). Moreover, in a work by Geisser et al. an optimal cut-off score of 21 for BDI was used to determine the presence of major depression (sensitivity 68. 2%, specificity 78. 4%) (7). Generally, the difference in depression occurrence between female and male in the current study turned out to be contrary to the result of a work by Asghari et al. conducted among Iranian patients with chronic pain in which there were no gender differences reported (8). And finally, Asghari and Nicholas concluded that the mean depression prevalence was 22% among Iranian patients with diagnosed chronic LBP taking into account 18 as the cut-off score of BDI (9). 

The mean decrease of BDI score after surgery reported by Farzanegan et al. is 9. 6 for female and 2. 7 for male (1). Because we do not know the baseline BDI score we are unable to recognize whether score changes (in percentages) are clinically noteworthy. But a great drop in BDI in female after surgery should be studied in detail. This is namely on the contrary to data reported by Sinikallio et al. , who found 1,66 decrease in BDI score 3 months after lumbar spinal stenosis surgery in comparison to the presurgical state (5). Additionally, all the result data in a study by Farzanegan et al. did not match control groups even by age and gender. Furthermore, the authors did not mention the mean depression-score at baseline and after surgery, whereas this data is also very important to compare the real differences in depression improvement by gender. The most important conclusion by Farzanegan et al. is the strong improvement in depression-score after surgery, especially among female patients (1). This indicates that the disability due to chronic low back pain is attributed to primary pain rather than to somatic symptoms of depression. This study may help those who are to make decision whether to operate or not on depressed patients with LBP, taking into account the fact that depression does not worsen the lumbar spinal surgery outcome. 
